# Global burden of cancer in women, 1990–2021: a systematic analysis from the GBD 2021 study

**DOI:** 10.3389/fonc.2025.1633894

**Published:** 2025-08-11

**Authors:** Yun Mao, Yebo Gao, Yongjie He, Zuihui Wan, Shuyu Li, Zhixun Ding, Beier Hu, Ling Fu, Chao Luo, Shijie Zhu, Wen Cao

**Affiliations:** ^1^ Department of Oncology, The Second Affiliated Hospital of Hunan University of Chinese Medicine, Changsha, Hunan, China; ^2^ Department of Oncology, Wangjing Hospital, China Academy of Chinese Medical Sciences, Beijing, China; ^3^ Nanfang Hospital, Southern Medical University, Guangzhou, China; ^4^ College of Information and Electrical Engineering, China Agricultural University, Beijing, China; ^5^ Department of Orthopedics and Traumatology, Huarong County Traditional Chinese Medicine Hospital, Yueyang, Hunan, China

**Keywords:** female cancers, Global Burden of Disease, breast cancer, cervical cancer, ovarian cancer, uterine cancer, global health equity

## Abstract

**Objective:**

This study evaluates global burden, disparities, and trends of female cancers (breast, cervical, uterine, ovarian) from 1990 to 2021, and identifies key contributing factors and intervention strategies.

**Methods:**

Data from the Global Burden of Disease (GBD,1990–2021) Study and recent reports were analyzed to assess incidence, mortality, and disability-adjusted life years (DALYs) across 204 countries stratified by Socio-demographic Index (SDI). Regression and spatiotemporal analyses explored links with risk factors (e.g., obesity, HPV) and healthcare access.

**Results:**

Breast cancer dominated the burden, with age-standardized incidence rates (ASIR) rising by 28% (2.08 million new cases in 2021), driven by lifestyle factors (high BMI, alcohol use) and showing a strong connection with higher SDI (r=0.82). Cervical cancer remained a critical challenge in low- and middle-income regions, showing a strong negative correlation with SDI(r = -0.75), though Age-Standardized Mortality Rate (ASMR) declined globally (-1.4% annual change). Uterine cancer incidence rose steadily (0.7% annual increase; 473,614 cases in 2021), primarily attributable to obesity, while ovarian cancer mortality remained high (207,000 deaths) due to late diagnosis. Key modifiable risks included HPV infection (85% of cervical cancers), tobacco use, and reproductive behaviors.

**Conclusion:**

The global burden of female cancers presents a significant public health challenge. Disparities in female cancer burden reflect inequities in healthcare access and rising metabolic risks. Priority actions include HPV vaccination, low-cost screening, and obesity control. Equity-focused, data-informed policies are crucial to reduce global disparities.

## 1 Introduction

The global burden of female cancers exhibits significant regional disparities and evolving epidemiology patterns. Breast cancer, the most prevalent cancer in women, accounted for 25% of all female cancer cases with 2.3 million new instances worldwide in 2022 (1). Projections suggest that, by 2050, breast cancer incidence will rise by 38% and mortality by 68% relative to 2022 levels (2). Cervical cancer remains a major public health challenge in sub-Saharan Africa and South America, where low- and middle-income countries (LMICs) account for 94% of global cervical cancer deaths, and exhibit a mortality rate of 12.4 per 100,000 population (3). This disproportionate burden is primarily attributable to with insufficient HPV vaccination coverage (<10% in many LMICs) and limited cervical screening implementation (<20% in high-burden regions). Gynecological malignancies demonstrate heterogeneous patterns across different levels of socioeconomic development (4). Uterine cancer incidence increased by 148% from 1990 to 2021, reaching 474,000 new cases in 2021, with high-income countries exhibiting an age-standardized incidence rate (ASIR) of 10.36 per 100,000 (5). Ovarian cancer, though less frequent (340,000 new cases in 2022), accounts for 50% of gynecological cancer mortality due to late-stage diagnosis and limited therapeutic advances (6).

There are significant disparities in female cancers across different regions and countries. The uneven distribution of medical resources has led to nearly one-third of breast cancer cases in sub-Saharan Africa being diagnosed at advanced stages at diagnosis, compared to only 5% in Europe and North America (7).Changes in lifestyle have introduced substantial health risks, with breast cancer accounting for over 40% of obesity-related malignancies. Studies indicate that each 5-unit increase in body mass index (BMI) is associated with a 12% increased risk of postmenopausal breast cancer, an effect more pronounced in Asian populations (8). Environmental factors contribute to the increasing burden of tumor-related diseases. For instance, the COVID-19 pandemic has severely disrupted global cancer control systems. During the pandemic in India in 2020, the missed diagnosis rate for tumors reached 83,600–111,500 cases, which is projected to cause an additional 98,650–131,500 cancer-related deaths over the next five years (9).

The Global Burden of Disease (GBD) Study database integrates comprehensive data spanning 1990 to 2021 across 204 countries and territories, encompassing disease incidence, mortality, disability-adjusted life years (DALYs), and exposure to 88 risk factors and injuries (10, 11). Leveraging a multidimensional analytical framework, this study systematically investigates the distribution patterns and temporal trends of female neoplasms across age strata, geographic regions (204 nations and 21 GBD subregions), and socioeconomic development levels, utilizing core metrics of incidence, mortality, and DALYs. These findings offer strategic insights for prioritizing resource allocation to achieve global cancer control targets, particularly in addressing disparities in healthcare access and optimizing early detection strategies.

## 2 Methods

### 2.1 Data acquisition

The data utilized in this study were obtained from the GBD 2021 database (https://vizhub.healthdata.org/gbd-results), a cutting-edge interdisciplinary platform integrating public health and data science. Our analysis extracted epidemiological indicators for breast, ovarian, uterine, and cervical cancers across 204 countries/territories and 21 super-regions. These metrics encompass annual incidence, prevalence, mortality, DALYs, and Age-Standardized Rates (ASR). Uncertainty quantification was implemented through Bayesian hierarchical modeling, generating 95% uncertainty intervals (UIs) from 1,000 posterior distribution samples (2.5thsutiono percentiles) to enhance statistical robustness.

The Socio-demographic Index (SDI) -a composite measure of national development–was calculated as the geometric mean of three standardized components: lag-distributed income per capita, average educational attainment among individuals aged ≥15 years, and total fertility rate under age 25. Following GBD stratification protocols, SDI values (range: 0-1) were categorized into five developmental tiers based on global population quartiles: high (0.805-1.000), high-middle (0.689iddle0), middle (0.608-0.689), low-middle (0.455-0.608), and low (0-0.455). This hierarchical classification enables systematic comparisons of health disparities across socioeconomic gradients (11).

### 2.2 Statistical analysis

This study utilized data from the GBD 2021 study to conduct a spatiotemporal trend analysis of the disease burden for four female-specific cancers (breast, ovarian, uterine, and cervical cancers) from 1990 to 2021. All disease burden metrics were reported per 100,000 population, including age-specific incidence and ASR. The ASR was calculated using the formula:


$$ASR=\left(\sum_{i=1}^{n}w_{i}.\frac{D_{i}}{P_{i}}\right)x10^{5}$$

Where D*i*represents the number of cases in the i-th age group, P*i*​denotes the corresponding person-years, and w*_i_* ​refers to the GBD standard population weights. Disease burden estimates incidence, mortality, and disability-adjusted life years, DALYs were reported as means with 95% uncertainty intervals (UI), derived from the 2.5th–97.5th percentiles of posterior distributions

For risk factor attribution, the population attributable fraction (PAF) was employed to quantify the proportion of deaths (%) and DALYs (%) attributable to specific risk factors, along with 95% UIs. The estimated annual percentage change (EAPC) and its 95% confidence interval (CI) were calculated using a natural log-linear regression model:


In(ASR)=α+β.Year+ϵ

where *β* is the regression coefficient, and EAPC=100×(exp(*β*)−1). Statistical significance was assessed via two-tailed *t*-tests (*p*<0.05) (12).

Nonlinear relationships between the SDI and cancer burden across 21 GBD regions and 204 countries/territories were modeled using locally weighted scatterplot smoothing (LOESS) (implemented via the geom_smooth function in R’s “ggplot2” package, smoothing parameter *span*=0.75). All statistical analyses and data visualizations were performed using R (version 4.4.2) and JD_GBDR (V2.37, Jingding Medical Technology Co., Ltd.). In this study, the R software package (version4.2.3) and JD_GBDR (V2.22, Jingding Medical Technology Co., Ltd.) was used for the drawing of the figures.

## 3 Results

### 3.1 Global, regional, and national burden of overall female cancers

Over the past three decades, breast cancer has persistently ranked as the most prevalent malignancy among women globally (**Table 1**). In 2021, the global incidence of breast cancer reached 2.08 million cases (95% UI: 1.94-2.23 million), significantly exceeding cervical (667,400; 95% UI: 613,030-726,422), uterine (473,600; 95% UI: 429,916-513,667), and ovarian cancers (298,900; 95% UI: 270,730-324,501). By comparison, 1990 incidence figures were substantially lower: breast cancer (865,900; 95% UI: 824,338-900,794), cervical (409,500; 95% UI: 383,207-438,506), uterine (191,300; 95% UI: 175,003-201,941), and ovarian cancers (159,100; 95% UI: 145,709–174,055). The ASIR in 2021 further emphasized this disparity, with breast cancer demonstrating the highest ASIR at 46.4 per 100,000 population (95% UI: 43.26–49.56), followed by cervical (15.32; 14.08-16.68), uterine (10.36; 9.42-11.24), and ovarian cancers (6.71; 6.07-7.28). Longitudinal analysis revealed divergent trends: breast and uterine cancers exhibited significant ASIR increases (EAPC 0.40 [95% CI: 0.35-0.45] and 0.54 [0.50-0.58], respectively) (**Supplementary Figures 1A, C**), while cervical and ovarian cancers showed declines (EAPC -0.54 [-0.64 to -0.44] and -0.38 [-0.43 to -0.32]), such as **Supplementary Figures 1B, D**.

**Table 1:** Global burden of female cancers in the year 1990 and 2021.

Variable	Breast cancer		Cervical cancer		Uterine cancer		Ovarian cancer	
	Age-standardized rate (per 100,000)	Number of cases	Age-standardized rate (per 100,000)	Number of cases	Age-standardized rate (per 100,000)	Number of cases	Age-standardized rate (per 100,000)	Number of cases
2021								
Incidence	46.4 (43.26,49.56)	2082737 (1940351,2225083)	15.32 (14.08,16.68)	667426 (613030,726422)	10.36 (9.42,11.24)	473614 (429916,513667)	6.71 (6.07,7.28)	298876 (270730,324501)
Prevalence	450.64 (427.02,475.96)	2082737 (1940351,2225083)	79.3 (72.81,86.58)	3384544 (3108399,3697258)	75.73 (69.37,81.78)	3451750 (3165257,3724779)	28.08 (25.26,30.64)	1222425 (1102106,1332112)
Deaths	14.55 (13.45,15.56)	660925 (609171,707182)	6.62 (6.07,7.18)	296667 (272059,321906)	2.11 (1.87,2.34)	97672 (86516,108062)	4.06 (3.67,4.4)	185609 (167962,201013)
DALYs	455.56 (426.64,485.3)	20254802 (18963376,21574429)	226.28 (206.51,246.86)	9911653 (9053317,10798306)	56.15 (50.07,62.37)	2562943 (2291154,2846493)	115.15 (104.58,125.21)	5163256 (4692423,5608304)
YLDs	32.2 (23.13,43.8)	1446370 (1040481,1968394)	6.54 (4.77,8.48)	282159 (206449,365881)	5.18 (3.74,6.94)	236511 (170823,316688)	3.51 (2.56,4.53)	155646 (113443,201143)
YLLs	423.36 (394.91,451.68)	18808432 (17539335,20067430)	219.75 (200.87,239.71)	9629494 (8809749,10511769)	50.97 (45.63,56.69)	2326432 (2084670,2586804)	111.64 (101.64,121.5)	5007611 (4561040,5448922)
1990								
Incidence	39.99 (38.01,41.60)	865881 (824338,900794)	18.11 (16.94,19.4)	409548 (383207,438506)	8.87 (8.12,9.35)	191291 (175003,201941)	7.22 (6.65,7.87)	159096 (145709,174055)
Prevalence	404.54 (377.02,439.95)	8689255 (8120449,9473540)	78.15 (73.89,82.51)	1826379 (1726564,1929080)	61.17 (56.35,64.14)	1333278 (1226415,1398593)	27.62 (25.26,30.26)	629090 (571666,693215)
Deaths	16.6 (15.60,17.45)	350577 (330510,368425)	9.68 (8.97,10.51)	211484 (195724,229841)	2.60 (2.32,2.80)	54849 (48804,59116)	4.73 (4.38,5.12)	100584 (92971,109087)
DALYs	503.81 (475.91,532.23)	11036402 (10434964,11671317)	330.11 (304.67,359.10)	7416287 (6841378,8071400)	69.17 (59.85,75.30)	1501433 (1297885,1637756)	132.48 (121.34,145.63)	2909236 (2662221,3199426)
YLDs	28.46 (20.45,37.95)	616131 (442046,822494)	7.01 (5.21,9.19)	160794 (119725,211438)	4.36 (3.18,5.82)	94670 (68926,126172)	3.66 (2.69,4.71)	81264 (59856,104604)
YLLs	475.35 (449.86,501.75)	10420271 (9863282,11010048)	323.1 (298.20,351.99)	7255493 (6694016,7905017)	64.81 (56.33,70.52)	1406762 (1220397,1533061)	128.82 (118.13,141.47)	2827972 (2589457,3106976)

DALYs, Disability-Adjusted Life Years; YLDs, Years Lived with Disability; YLLs, Years of Life Lost.

Mortality patterns demonstrated similar complexity, with breast cancer deaths increasing from 350,600 (1990) to 660,900 (2021) yet showing declining ASMR (EAPC -0.55 [-0.60 to -0.50]), reflecting therapeutic advancements. Cervical cancer mortality decreased from 350,600 to 296,700 (EAPC -1.27 [-1.37 to -1.18]), likely attributable to HPV vaccination and screening, while uterine cancer deaths surged from 26,000 to 97,700 despite ASMR reductions (EAPC -0.78 [-0.85 to -0.70]). Ovarian cancer mortality nearly doubled (100,600 to 185,600) with gradual ASMR decline (EAPC -0.62 [-0.68 to -0.57]). These epidemiological shifts underscore the dual challenges of population growth and aging against progress in cancer control, highlighting the urgent need for targeted prevention strategies and therapeutic innovations to address persistent disparities in women’s cancer burden.

### 3.2 Regional disparities in the burden of female cancers

**Figure 1:**
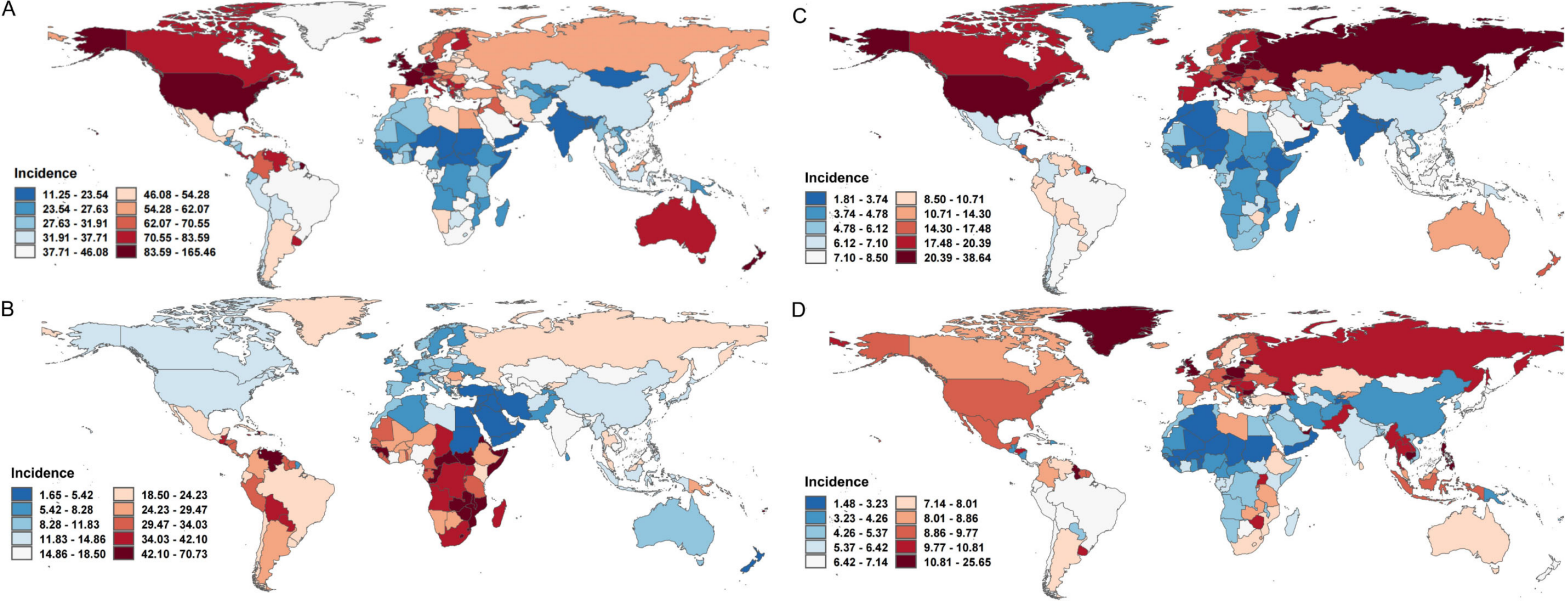
The ASIR for female cancers in 204 countries, 2021. **(A)** The ASIR for breast cancers of 204 countries in 2021; **(B)** The ASIR for cervical cancers of 204 countries in 2021; **(C)** The ASIR for uterine cancers of 204 countries in 2021; **(D)** The ASIR for ovarian cancers of 204 countries in 2021. ASIR, age-standardized incidence rate.

In 2021, breast cancer exhibited the ASIR in High-income North America (94.93 per 100,000; 95% CI: 89.02-98.93), while South Asia had the lowest ASIR (24.62; 21.52-28.32) (**Supplementary Table 1**). Antigua and Barbuda, Greece, and Jamaica reported the highest national incidence rates, whereas China recorded the largest number of new cases (385,838), followed by the United States (269,012) and India (156,160). From 1990 to 2021, ASIR trends rose globally, with North Africa and the Middle East showing the steepest increase (EAPC=4.04; 95% CI:3.80-4.28). High-income regions consistently had the highest ASIR (77.08; 71.83-79.93), contrasting with low-income regions (24.09; 21.34-26.87). Breast cancer mortality declined from 16.60 to 14.55 per 100,000 during this period, yet deaths nearly doubled (350,577-660,925), driven by demographic shifts. Southern Sub-Saharan Africa had the highest mortality rate (24.93; 22.64-27.46), while South Asia reported the most deaths (105,497), with China (88,107), India (78,879), and the U.S. (52,869) bearing the highest national burdens (**Figure 1A**, **Table 2**).

**Table 2:** ASIR and ASMR of female cancers with low to high SDI in 2021.

Location	Breast cancer		Cervical cancer		Uterine cancer		Ovarian cancer	
	ASIR (per 100 000)	ASMR (per 100 000)	ASIR (per 100 000)	ASMR(per 100 000)	ASIR (per 100 000)	ASMR(per 100 000)	ASIR (per 100 000)	ASMR(per 100 000)
Global	46.4 (43.26,49.56)	14.55(13.45,15.56)	15.32(14.08,16.68)	6.62 (6.07,7.18)	10.36(9.42,11.24)	2.11 (1.87,2.34)	6.71 (6.07,7.28)	4.06 (3.67,4.4)
High SDI	77.08 (71.83,79.93)	15.44(14.01,16.21)	10.3 (9.91,10.66)	2.62 (2.44,2.74)	19.55(18.28,20.35)	2.69 (2.41,2.84)	8.4 (7.84,8.81)	5.08 (4.62,5.37)
High-middle SDI	51.05 (46.06,57.18)	13.87(12.63,15.13)	13.27(11.44,15.16)	4.59 (4.02,5.2)	14.23(12.89,15.75)	2.39 (2.13,2.66)	6.93 (6.1,7.76)	4.16 (3.68,4.63)
Middle SDI	37.16 (33.42,41.13)	12.68 (11.44,14.1)	15.94 (14.3,17.75)	6.72 (6.05,7.43)	6.09 (4.97,7.2)	1.61 (1.35,1.87)	5.81 (5.07,6.5)	3.18 (2.78,3.57)
Low-middle SDI	28.29 (25.52,30.93)	14.59(13.07,16.04)	17.79 (15.94,19.7)	9.71 (8.67,10.7)	4.13 (3.56,5.08)	1.64 (1.42,2.07)	6.01 (5.22,7.07)	3.72 (3.24,4.4)
Low SDI	24.09 (21.34,26.87)	16(14.15,17.91)	25.47(21.57,30.12)	16.36(13.94,19.38)	3.58 (2.87,4.59)	1.83 (1.46,2.36)	5.42 (4.12,6.43)	3.74 (2.87,4.43)

ASIR, age-standardized incidence rate; ASMR, age standardized mortality rate; SDI, Socio-demographic index.

Cervical cancer ASIR decreased globally from 19.13 (95% UI: 17.50-20.19) in 1990 to 15.32 (14.08-16.68) in 2021, inversely correlating with socioeconomic development (High SDI: 10.3 vs. Low SDI: 25.47) (**Supplementary Table 1**, **Figure 1B**). Central Sub-Saharan Africa had the highest regional ASIR (42.4; 37.16-47.85), whereas North Africa and the Middle East reported the lowest (4.72; 4.04-5.50). Mortality trends paralleled incidence declines (Age-Standardized Mortality Rate [ASMR] EAPC =-1.27), though low SDI regions remained disproportionately affected, with Central Sub-Saharan Africa having the highest mortality rate (25.1; 17.45-33.97) and South Asia the most deaths (703,100).

Uterine cancer, the fastest-growing female malignancy, saw global ASIR rise at an EAPC of 0.54 (0.50-0.58), with High-income North America (31.78; 29.88-33.21) and Eastern Sub-Saharan Africa (4.1; 3.00-5.59) representing the highest and lowest regional rates, respectively (**Supplementary Table 1**, **Figure 1C**). ASMR declined modestly (EAPC =-0.78), with the Caribbean having the highest mortality rate (5.38; 4.63-6.24) and East Asia the most deaths (14,233), predominantly in China (13,599).

Ovarian cancer, with a global ASIR of 6.71 (6.07-7.28) in 2021, showed rising trends in Low-middle SDI regions (EAPC = 1.51) (**Supplementary Table 1**, **Figure 1D**). Central Europe had the highest regional ASIR (10.8; 9.93-11.68), while China reported the most cases (41,236). ASMR slightly declined (EAPC =-0.62), with Central Europe having the highest ASMR(7.4) and South Asia the largest death toll (30,585). These trends underscore persistent disparities in cancer burden, emphasizing the need for region-specific prevention and control strategies aligned with socioeconomic and epidemiological contexts.

**Figure 2:**
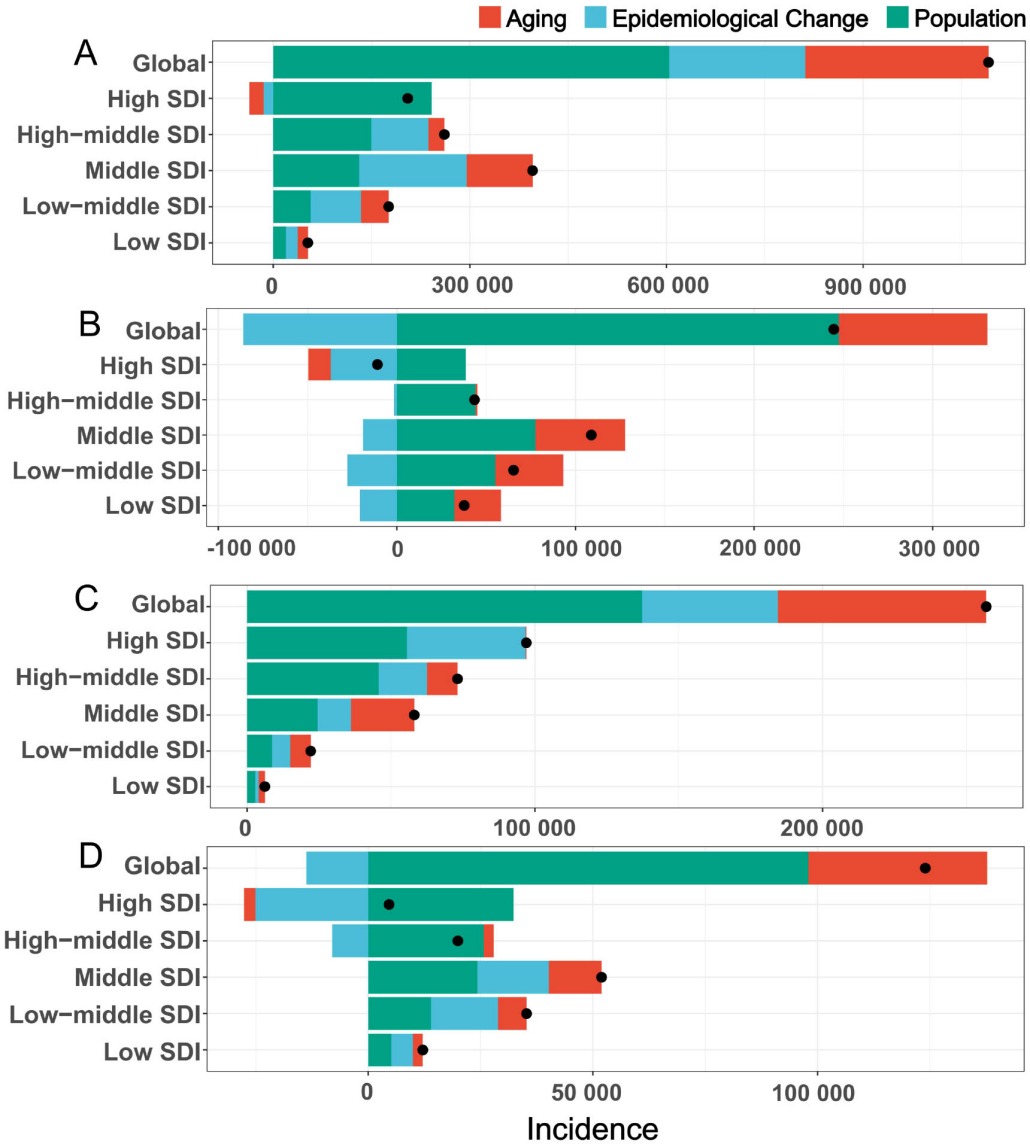
The decomposition analysis of global female tumor incidence from 1990 to 2021. **(A)** breast tumor; **(B)** cervical tumor; **(C)** uterine tumor; **(D)** ovarian tumor. Black dots represent the overall changes in disease burden due to aging, epidemiological changes, and population growth. For each component, an increase in the disease burden of tumor related to that component is indicated by positive values, whereas a decrease is indicated by negative values. SDI, socio-demographic index.

The research team conducted a decomposition analysis on breast cancer, cervical cancer, endometrial cancer, and ovarian cancer to assess the contributions of population growth, aging, and epidemiological changes to their global incidence trends (**Figure 2**, **Supplementary Table 2**). For breast cancer, population growth accounted for 55.37% of the global incidence increase, followed by aging(25.62%) and epidemiological changes (19.01%); however, in High SDI regions, population growth contributed 117.9% (offset by a -10.72% decline from aging), while epidemiological changes dominated in Middle and Low-middle SDI regions(41.44% and 43.66%, respectively). For cervical cancer, population growth contributed 101.12% globally, with aging (34.04%) and biological factors (-35.16%, e.g., HPV prevention) playing contrasting roles; in High SDI regions, epidemiological changes (e.g., vaccination programs) drove a 340.15% increase despite a -355.1% offset from population decline. Endometrial cancer incidence growth was primarily linked to population growth (53.44%), aging (28.2%), and epidemiological changes (18.36%), with the latter’s adverse effects diminishing at lower SDI levels. For ovarian cancer, population growth contributed 79.02% globally in 2021, alongside aging (32.1%) and epidemiological changes (-11.12%); economic development amplified demographic impacts, with population growth contributing 42.25% in Low SDI regions versus 698.64% in High SDI regions, likely due to delayed reproductive patterns. These findings highlight significant regional disparities in cancer burden drivers, emphasizing the interplay of demographic shifts, socioeconomic development, and evolving risk factors.

### 3.3 Burden of female cancers by SDI

**Figure 3:**
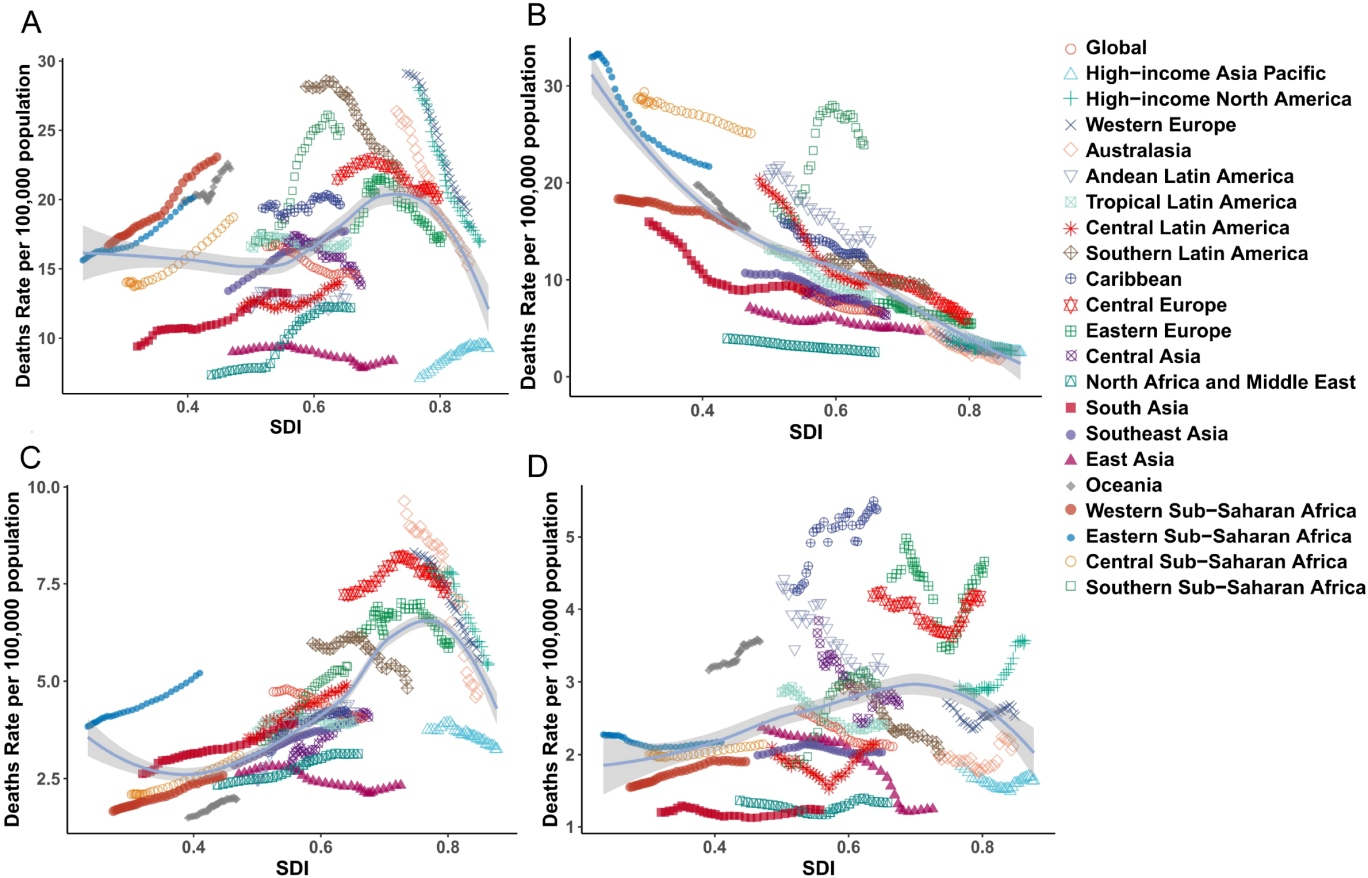
The trends of ASMR for female cancer from 1990 to 2021, by SDI groups across 21 regions and globally. **(A)** breast tumor; **(B)** cervical tumor; **(C)** uterine tumor; **(D)** ovarian tumor. ASMR, age standardized mortality rate; SDI, socio-demographic index.

The SDI serves as a comprehensive indicator for evaluating regional development levels. Between 1990 and 2021, regions across varying SDI categories (excluding high SDI regions) demonstrated upward trends in the ASIR, age-standardized prevalence rate (ASPR), and ASMR, and for breast cancer, uterine cancer, and ovarian cancer (**Figure 3**). In contrast, cervical cancer exhibited a negative correlation with SDI across all four metrics. Notably, high SDI regions, including Western Europe, Central Europe, high-income North America, and Australasia, displayed declining patterns in ASIR, ASPR, ASMR, and ASDR for these cancers. By 2021, this divergence persisted: breast, uterine, and ovarian cancers maintained positive correlations with SDI as regions developed economically, while high SDI regions remained exceptions to this trend, continuing to show reductions in disease burden metrics. This phenomenon highlights the complex interplay between socioeconomic development and cancer epidemiology, particularly the distinct trajectory of cervical cancer compared to hormone-sensitive cancers like breast, uterine, and ovarian malignancies.

### 3.4 Attributable burden of female cancers caused by risk factors

**Figure 4:**
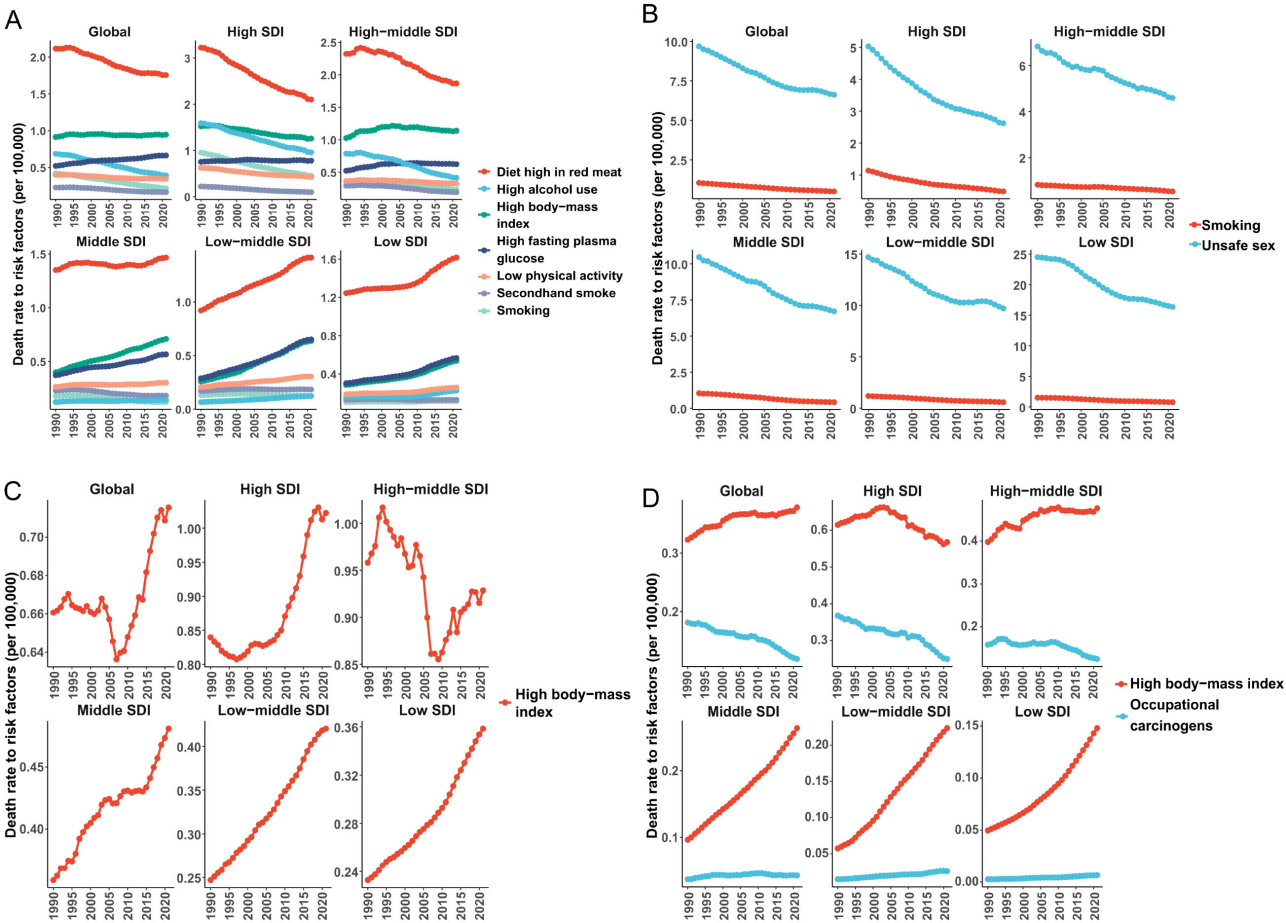
The contribution and changing trends of female cancer risk factors to ASMR from 1990 to 2021. **(A)** breast tumor; **(B)** cervical tumor; **(C)** uterine tumor; **(D)** ovarian tumor. ASMR, age standardized mortality rate; SDI, socio-demographic index.

According to the GBD 2021 data, the attributable risks for breast cancer, cervical cancer, uterine cancer, and ovarian cancer exhibit distinct epidemiological patterns. For breast cancer (**Figure 4A**), seven primary risk factors were identified: smoking (0.22 per 100,000 population), secondhand smoke (0.16 per 100,000), high body-mass index (BMI, 0.95 per 100,000), high alcohol use (0.39 per 100,000), diet high in red meat (1.76 per 100,000), high fasting plasma glucose (0.66 per 100,000), and low physical activity (1.76 per 100,000). Globally, the ASMR attributable to diet high in red meat remained the highest contributor to breast cancer mortality, though it showed a slight downward trend. Notably, high and high-middle SDI regions demonstrated declining ASMR for red meat-related breast cancer deaths over the past three decades, while low SDI regions experienced increasing trends. Concurrently, the other six risk factors exhibited rising ASMR in low, middle, and low-middle SDI regions.

Cervical cancer mortality was predominantly driven by unsafe sexual behavior across all SDI categories, with this factor contributing an ASMR reduction from 9.68 (95% UI 8.96-10.52) in 1990 to 6.62 (95% UI 13.94-19.38) in 2021 (**Figure 4B**). Tobacco use emerged as a secondary but persistent risk factor, particularly in high SDI regions. For uterine cancer (**Figure 4C**), high BMI stood as the sole Level 2 risk factor, with its ASMR increasing globally from 2008 to 2021 (reaching 0.72 per 100,000 in 2021). This upward trajectory was most pronounced in low SDI regions, reflecting accelerated adiposity-related epidemiological transitions in developing economies. Ovarian cancer mortality showed dual attribution to high BMI and occupational hazards. High BMI accounted for the majority of ASMR burden, particularly in low- and middle-income SDI regions where its contribution increased steadily (**Figure 4D**). In contrast, occupational risks exhibited divergent trends: high-income regions demonstrated declining ASMRs for occupation-related ovarian cancer, while middle SDI regions maintained stable patterns. These findings underscore the critical need for SDI-stratified prevention strategies, emphasizing dietary modifications for breast cancer, HPV vaccination and smoking cessation for cervical cancer, weight management for uterine cancer, and occupational safety regulations combined with metabolic interventions for ovarian cancer.

### 3.5 Attribution analysis of risk factors in different regions and ages of female tumors

#### 3.5.1 Breast cancer

Researchers further analyzed the attributable risk differences across 21 global regions and various age groups in female breast cancer mortality. The study revealed that smoking, secondhand smoke exposure, high body-mass index (BMI), high alcohol use, diet high in red meat, high fasting plasma glucose, and low physical activity were significant risk factors contributing to increased breast cancer mortality (**Supplementary Figure 2**). In 2021, a diet high in red meat emerged as the predominant risk factor, accounting for 6.6% to 13.7% of attributable mortality risk across the 21 regions, with the lowest impact observed in South Asia and the highest in Australasia. High alcohol use exhibited substantial regional variability, contributing to elevated mortality risks in Australasia (8.4%), Western Europe (7.5%), and High-income Asia Pacific (6.3%). Other risk factors showed no significant regional variations across the 21 regions.

Age-stratified analyses demonstrated distinct patterns: smoking, secondhand smoke, and high alcohol use caused mortality risk increments of 1%-3%, 1%-2%, and 1%-3%, respectively, with no marked age-related disparities (**Supplementary Figure 3**). High BMI did not elevate mortality risk in individuals aged<50 years but significantly increased it (8.6%-9.3%) in those ≥50 years. Conversely, diet high in red meat, high fasting plasma glucose, and low physical activity exhibited age-progressive effects, with attributable risks rising incrementally across older age groups. Among individuals aged ≥80 years, these three factors contributed 12.8%, 5.7%, and 3.7% of breast cancer mortality risk, respectively, in 2021. These findings underscore the critical interplay of modifiable lifestyle factors and age-specific biological mechanisms in breast cancer mortality, highlighting the necessity for regionally tailored and age-stratified prevention strategies.

#### 3.5.2 Cervical cancer

The risk factors for cervical cancer include unsafe sexual behaviors and alcohol consumption (**Supplementary Figure 4**). Across 21 distinct regions and various age groups, unsafe sexual behaviors consistently emerge as the predominant risk factor for cervical cancer mortality, with no significant regional or age-related disparities observed. In contrast, the impact of alcohol consumption exhibits substantial regional variations. Notably, regions such as High-income North America (22.5%), Western Europe (21.7%), and Southern Latin America (23.4%) demonstrate alcohol-related mortality risk increases exceeding 20%. Regarding smoking, its association with cervical cancer mortality varies significantly across age groups. Specifically, smoking does not elevate mortality risk in individuals under 30 years of age, whereas it becomes a contributory factor in older demographics (**Supplementary Figure 5**). These findings underscore the universal significance of sexual health interventions while highlighting the need for region-specific strategies targeting alcohol consumption and age-tailored smoking cessation programs.

#### 3.5.3 Uterine cancer

High BMI is identified as the sole risk factor contributing to increased mortality risk from uterine cancer, such as **Supplementary Figure 6**. The population attributable fraction of uterine cancer deaths linked to elevated BMI exhibits significant regional heterogeneity, with Western Europe, Australasia, and High-income North America demonstrating markedly higher risk burdens compared to other geographic regions. Furthermore, age-stratified analyses reveal a progressive escalation in mortality risk associated with excess BMI across successive age cohorts. This risk trajectory peaks within the 60–64 years age group, where 36.2% of uterine cancer deaths are attributable to elevated BMI-the highest proportion observed across all demographic segments (**Supplementary Figure 7**). The differential regional risk profiles and age-dependent risk amplification underscore the critical interplay between metabolic derangements, population-specific lifestyle patterns, and biological aging processes in shaping uterine cancer outcomes.

#### 3.5.4 Ovarian cancer

In the context of ovarian cancer, high BMI and occupational risk factors are identified as predominant contributors to mortality(**Supplementary Figure 8**). Elevated BMI consistently increases ovarian cancer mortality risk across all 21 analyzed regions, with the North Africa and Middle East region exhibiting a 16.3% rise in mortality risk. Furthermore, the contribution of elevated BMI to ovarian cancer mortality escalates progressively with advancing age. Occupational risk factors, however, demonstrate marked regional heterogeneity. In 2021, these factors significantly heightened ovarian cancer mortality in Australasia (9.8%) and Western Europe (7.1%), while their impact remained statistically non-significant in other regions. Age-stratified analyses reveal that occupational exposures do not substantially elevate mortality risk in individuals under 35 years, whereas in populations aged 35 and above, the mortality risk attributable to occupational factors exhibits a progressive age-dependent increase, reaching 7.5% in those over 80 years old(**Supplementary Figure 9**). These findings underscore the dual influence of metabolic and environmental determinants on ovarian cancer outcomes, with age modulating both biological susceptibility and cumulative exposure duration.

### 3.6 Projections of the future global burden of female cancers

**Figure 5:**
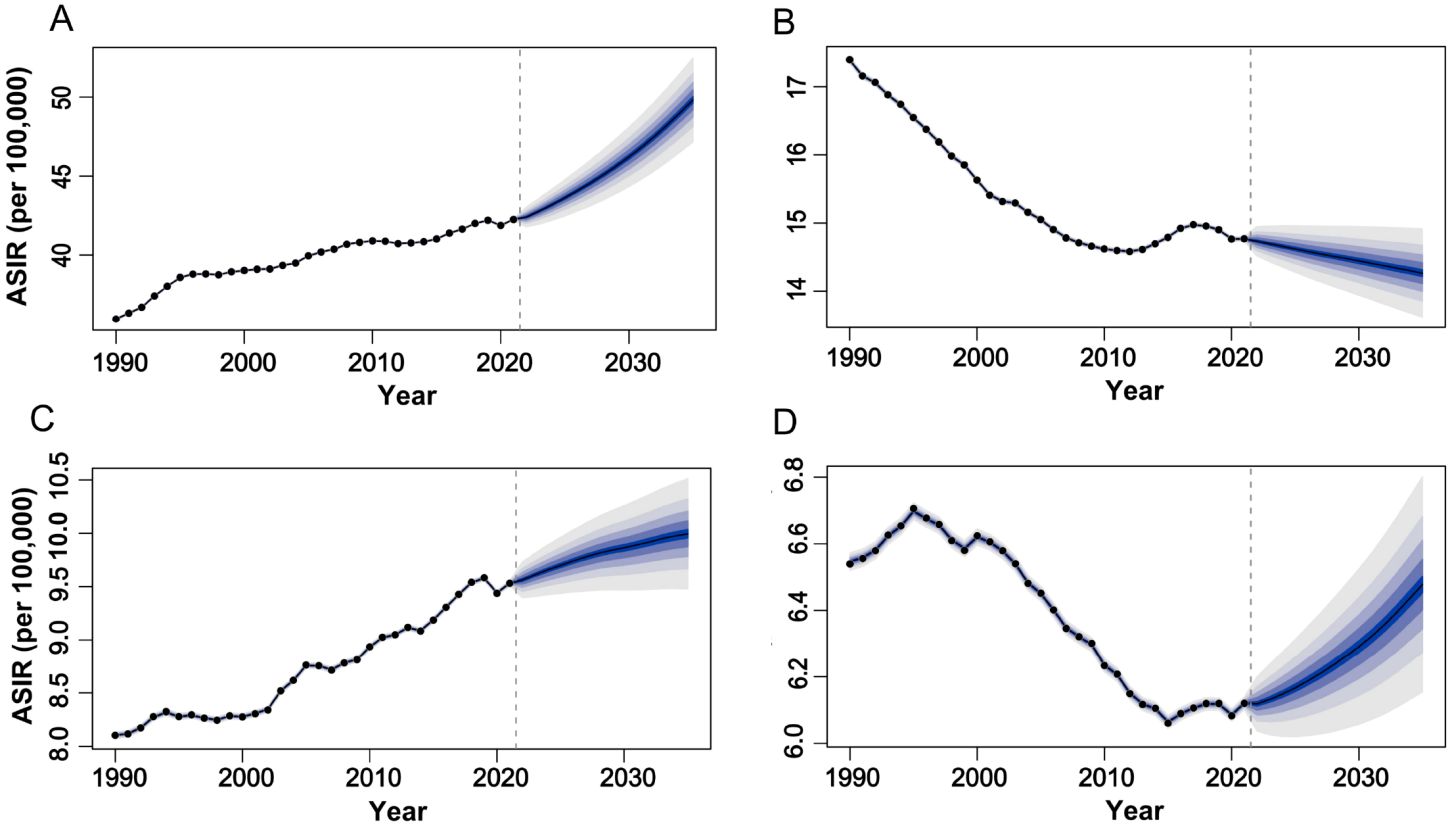
The global trends in ASIR of female tumors from 1990 to 2035. **(A)** breast tumor; **(B)** cervical tumor; **(C)** uterine tumor; **(D)** ovarian tumor. ASIR, age-standardized incidence rate.

Based on projections from the Bayesian Age-Period-Cohort (BAPC) model, which integrates age, period, and cohort effects using full Bayesian inference with integrated nested Laplace approximations, the epidemiological trends for female-specific cancers from 2022 to 2035 reveal distinct patterns (**Figure 5**). For breast cancer (**Figure 5A**), the ASIR is projected to gradually increase, reaching 49.83 per 100,000 population by 2035, while the ASMR is expected to rise slightly to 12.25 (95% CI: 11.61tlyonci in the same period. In contrast, uterine and ovarian cancers exhibit divergent trends: uterine cancer ASIR is anticipated to climb to 10.0 (95% CI: 6.09-6.83) by 2035, and ovarian cancer ASIR is projected to rise to 6.47 (95% CI: 9.38-10.61), whereas their mortality rates show declines, with uterine cancer ASMR decreasing to 1.40 (95% CI: 1.28-1.51) and ovarian cancer ASMR dropping to 3.31 (95% CI: 3.12-3.48) by 2035 (**Figures 5C**, D). Cervical cancer demonstrates sustained reductions in both incidence and mortality, with ASIR forecasted to decline from 14.73 (95% CI: 14.48-14.99) in 2021 to 14.26 (95% CI: 14.49-15.04) in 2035, and ASMR slightly decreasing from 5.93 (95% CI: 5.81-6.06) to 5.15 (95% CI: 4.81-5.49) per 100,000 population (**Figure 5B**). These trends may reflect evolving risk factors such as lifestyle changes, delayed childbirth, and advancements in screening for breast, uterine, and ovarian cancers, alongside the success of HPV vaccination programs and early detection efforts in reducing cervical cancer burden. Mortality declines for uterine and ovarian cancers further suggest progress in therapeutic interventions, underscoring the need for targeted prevention and treatment strategies.

## 4 Discussion

This study utilized the GBD database to systematically elucidate global epidemiological evolution trends of breast, cervical, uterine, and ovarian cancers, revealing multidimensional heterogeneity in incidence, mortality, and risk characteristics. These cancers collectively account for approximately 40% of the total female cancer burden, demonstrating divergent trends due to differences in regional development levels, modifiable exposure factors, and healthcare accessibility (2). This study analyzed disparities in female malignancies across global and regional dimensions from 1990 to 2021. In 2021, breast cancer demonstrated the highest ASIR, DALYs, and ASMR among female malignancies. From 1990 to 2021, breast and uterine cancers showed continued upward trends in ASIR, while cervical and ovarian cancers exhibited declining ASIR trends; meanwhile, the ASMR for all four cancer types consistently decreased. This research provides a comprehensive assessment of the global burden of female-specific cancers, aiming to lay the groundwork for healthcare service planning and interventions.

Breast cancer epidemiological characteristics demonstrate close association with socioeconomic development (13). In 2021, 2.08 million new breast cancer cases were reported globally, with substantial geographical disparities. High SDI regions such as North America exhibited an ASIR of 95.1 per 100,000 population, more than three times that of low-SDI regions like South-central Asia (26.67 per 100,000), with North America and Western Europe consistently exceeding the global average (2). However, high-SDI regions demonstrated a decline in ASMR from 16.60 per 100,000 in 1990 to 14.55 per 100,000 in 2021 (2). Age distribution analysis shows that 71% of global cases occur in individuals ≥50 years old, whereas 47% of new cases in sub-Saharan Africa concentrate in those<50 years (14), which is associated with multiple factors including the region’s high proportion of young population, elevated BRCA mutation frequency, insufficient screening, and diagnostic delays (15, 16). Notably, China experienced an annual incidence growth rate of 3.7% among women<40 years, significantly higher than the 2% observed in older populations, with both ASIR and ASMR exhibiting marked age-dependent increases (17).

Risk attribution analysis confirms that smoking, secondhand smoke, high BMI, alcohol use, red meat consumption, elevated fasting plasma glucose, and physical inactivity are the main death drivers of breast cancer, all of which are associated with modern lifestyle and urbanization (18). Diet high in red meat contributed to the highest ASMR among these factors, though with a slight downward trend. High-SDI regions were predominantly influenced by alcohol consumption (population attributable fraction: 21.3%) and red meat intake (19, 20), While low-SDI regions faced elevated risks from secondhand smoke exposure (>30% increased risk) and alcohol use. These disparities reflect socioeconomic contexts: rising meat consumption parallels wealth accumulation in high-SDI countries (21), Whereas inadequate tobacco control policies and high male smoking rates exacerbate passive smoking risks among young women in low-SDI areas (22). Intriguingly, high BMI may confer protective effects against breast cancer in young women via ovarian hormone suppression. Prevention strategies require regional adaptation: high-SDI regions should prioritize alcohol and red meat consumption controls (23), while low-SDI areas require enhanced public no-smoking policies and health education to improve risk awareness. Despite a 1.2% annual mortality reduction in high-income countries through targeted therapies like CDK4/6 inhibitors (e.g., palbociclib, ribociclib, abemaciclib), low- and middle-income regions face challenges due to inadequate trastuzumab coverage (<5%, with annual costs equivalent to 300% of per capita GDP), underscoring the urgent need for globally differentiated resource allocation and risk intervention strategies (24–26).

Cervical cancer, as the only malignancy with well-defined etiology, exhibits a disease burden closely correlated with the accessibility of HPV vaccination and screening programs (27). The global ASIR is declining at an annual rate of 1.5%, yet significant regional disparities persist: Sub-Saharan Africa exhibits an ASIR as high as 27.5 per 100,000 (compared to merely 4.6 per 100,000 in Western Europe), with 94% of deaths concentrated in LMICs, a phenomenon closely linked to inadequate resources for screening, vaccination, and treatment in these areas (28, 29). Age-specific analyses reveal a notable ASIR reduction of 2.2% per annum among women under 50 years, though low-income areas face persistent challenges with HPV vaccination coverage below 5% in young females, perpetuating high incidence rates (30). Primary risk factors encompass unsafe sexual practices (multiple sexual partners, early sexual debut) and tobacco use, with persistent infection by high-risk HPV types 16 and 18 being the principal carcinogenic drivers (31, 32). The three-tiered prevention framework demonstrates substantial efficacy: Countries achieving >70% coverage of 9-valent HPV vaccination (e.g., Australia) report 90% reductions in precancerous lesions (33), and HPV self-sampling techniques elevate screening participation to 60% in resource-limited settings (34). Immunotherapeutic advancements, particularly pembrolizumab, have extended the median survival time of metastatic cervical cancer patients by 10.35 months (35). Yet, global HPV vaccine coverage remains critically low at 12.2% (2018 data), compounded by 41% treatment discontinuation rates in Central Africa, underscoring systemic fragility in healthcare delivery (36, 37). Smoking increases cervical cancer risk 2–3 fold and worsens lesion severity in a dose-dependent manner, with synergistic interactions between smoking and HPV persistence accelerating progression to cervical intraepithelial neoplasia grade 3 or higher (32, 38). These insights underscore the need for integrated behavioral and biomedical prevention strategies.

Uterine cancer, a metabolic syndrome-driven “disease of affluence”, exhibits a strong positive correlation between its ASIR and SDI, with high-SDI countries reporting an ASIR of 10.36 per 100,000 compared to 3.23 per 100,000 in low-SDI countries. Across all age groups, individuals aged 60–80 years account for 65% of cases, while a concerning annual growth rate of 4.7% in ASIR is observed among young females (<50 years), closely associated with obesity (BMI ≥30 elevates risk by 300%) (39). In high-income regions such as North America, dietary patterns characterized by fat contributing >35% of total energy intake and sedentary lifestyles (prolonged sedentary behavior >6 hours/day increases risk by 40%) have established these areas as epidemiological hotspots, where high BMI accounts for 38.7% of population-attributable risk. Prevention strategies require dual approaches: high-SDI regions should prioritize GLP-1 receptor agonists (e.g., semaglutide) demonstrating 18% risk reduction in obesity-related cases, alongside minimally invasive surgical techniques that reduce complication rates by 50% (40). Conversely, low-SDI regions necessitate enhanced community-based diabetes management protocols and standardized BMI surveillance systems to mitigate emerging risks.

Ovarian cancer is characterized by its insidious nature and high mortality rate, with 70% of patients diagnosed at an advanced stage (5-year survival rate<30%), and BRCA1/2 mutation carriers facing a lifetime risk of 44%-17% (41, 42). High-SDI countries have achieved an annual average 0.9% decline in ASDR through widespread adoption of PARP inhibitors such as olaparib (utilization rate >60%), whereas middle- and low-SDI regions experience a 0.7% annual ASDR increase due to genetic testing coverage below 5% (43). Elevated BMI and occupational exposures remain major risk factors for ovarian cancer mortality, with multiple large-scale studies confirming a 6%-10% increase in ovarian cancer risk per 5 kg/m² BMI increment (44). This is especially pronounced in women not using hormone replacement therapy, where postmenopausal estrogen from adipose tissue promotes carcinogenesis (45). Therapeutic innovations like hyperthermic intraperitoneal chemotherapy have extended median survival by 16 months in advanced-stage patients (46), yet global disparities in pathologist distribution (4.5 per 100,000 population in North America vs. 0.1 per 100,000 in Sub Saharan Africa) significantly constrain diagnostic and therapeutic capabilities (47). Future strategies necessitate establishing regional BRCA mutation databases (e.g., the African VUS-AC1 type) to guide precision interventions such as risk-reducing salpingectomy (48).

Our findings reveal divergent trends in the incidence of female-specific cancers across socio-demographic strata. Specifically, the rising ASIR of breast and endometrial cancers in high-SDI regions appears to be driven by increasing obesity prevalence and shifting reproductive behaviors, including delayed childbearing, reduced parity, and shorter breastfeeding duration—all of which are recognized hormonal and metabolic risk factors. These trends are further compounded by lifestyle changes such as physical inactivity and high-fat dietary patterns. In contrast, the declining ASIR of cervical cancer observed in some low- and middle-income countries may reflect the early impact of modest HPV vaccination uptake and enhanced awareness of cervical cancer prevention (49). Nonetheless, this encouraging trend coexists with persistent screening limitations, especially in rural and underserved populations where cytology-based or HPV DNA testing remains inaccessible or underutilized. These patterns underscore the dual burden of epidemiological transition: rising hormone- and lifestyle-related cancer incidence in high-SDI settings and ongoing structural barriers to infectious cancer control in resource-limited regions. Overall, this study provides a comprehensive, SDI-stratified analysis of female cancers over three decades, identifying key regional disparities and risk factor trends. These findings support evidence-based decision-making for national cancer prevention and control programs, facilitating development of equity-focused precision strategies. Simultaneously, the GBD database analysis of female cancers demonstrates certain limitations including uneven regional coverage (cancer registries in low-income countries<5%), missing subtype information (e.g., HPV typing and molecular subtypes of breast cancer), and high model dependency (extrapolation increases uncertainty). Regarding attributional causality inference, challenges include incomplete coverage of risk factors (omitting key variables like genetics and hormones) and confounding factor interference, necessitating strategies like enhancing data quality and stratified disease burden reporting.

## 5 Conclusions

To address these challenges, current prevention strategies must implement region-specific interventions. In high sociodemographic index (SDI) regions (>0.81), priorities should focus on enhancing metabolic disease management (given that obesity-associated breast cancer accounts for over 40% of cases) and improving access to targeted therapies (with ovarian cancer DALYs reaching 5.16 million). In low and middle-income regions, immediate actions should focus on adopting single-dose HPV vaccination regimens, which offer 70% cost savings and 90% efficacy, alongside establishing community-based screening systems. HPV self-sampling methods could help increase cervical cancer screening coverage to 60% in preserved populations. A global collaborative mechanism modeled after the COVAX vaccine procurement alliance is critical for reducing HPV vaccine costs from $50 per dose to $4.5 per dose in resource-limited settings. Technological advancements, such as AI-assisted mammography—using neural networks like TMuNet and adaptive normalization algorithms—have the potential to lower false-negative rates by 40% and improve diagnostic capacity in low-resource areas (50). The WHO’s 2030 cervical cancer elimination target (ASIR<4 per 100,000) is being validated through multinational initiatives, exemplified by Brazil’s 60% cervical cancer mortality reduction via its Unified Health System and China’s Ordos pilot program achieving 90% HPV vaccination coverage through government-funded immunization (51). Updated GBD 2021 data reveal diverging trends: while breast cancer DALY rates declined annually by 0.73% from 1990-2021, uterine cancer ASIR increased by 0.16% annually, necessitating region-specific prioritization. Central Latin America requires intensified breast cancer early detection (ASIR 46.5/100,000) through organized population-based screening programs, whereas sub-Saharan Africa must prioritize cervical cancer control (DALY rates exceeding 300/100,000) via integrated HPV vaccination and VIA/VILI screening networks. By synergizing tiered screening optimization, vaccine policy innovation, and technological empowerment, a 50% reduction in breast cancer disparities across regions could be achieved by 2030, establishing a concrete pathway toward global health equity for women.

## Data Availability

The original contributions presented in the study are included in the article/**Supplementary Material**. Further inquiries can be directed to the corresponding authors.
