# Supplementary material for: Global burden of cancer in women, 1990–2021: a systematic analysis from the GBD 2021 study
Source: Front Oncol. 2025 Aug 11;15:1633894. doi: 10.3389/fonc.2025.1633894 (PMC12376056; doi:10.3389/fonc.2025.1633894)
Supplement: Supplementary file 1 [file DataSheet1.docx]

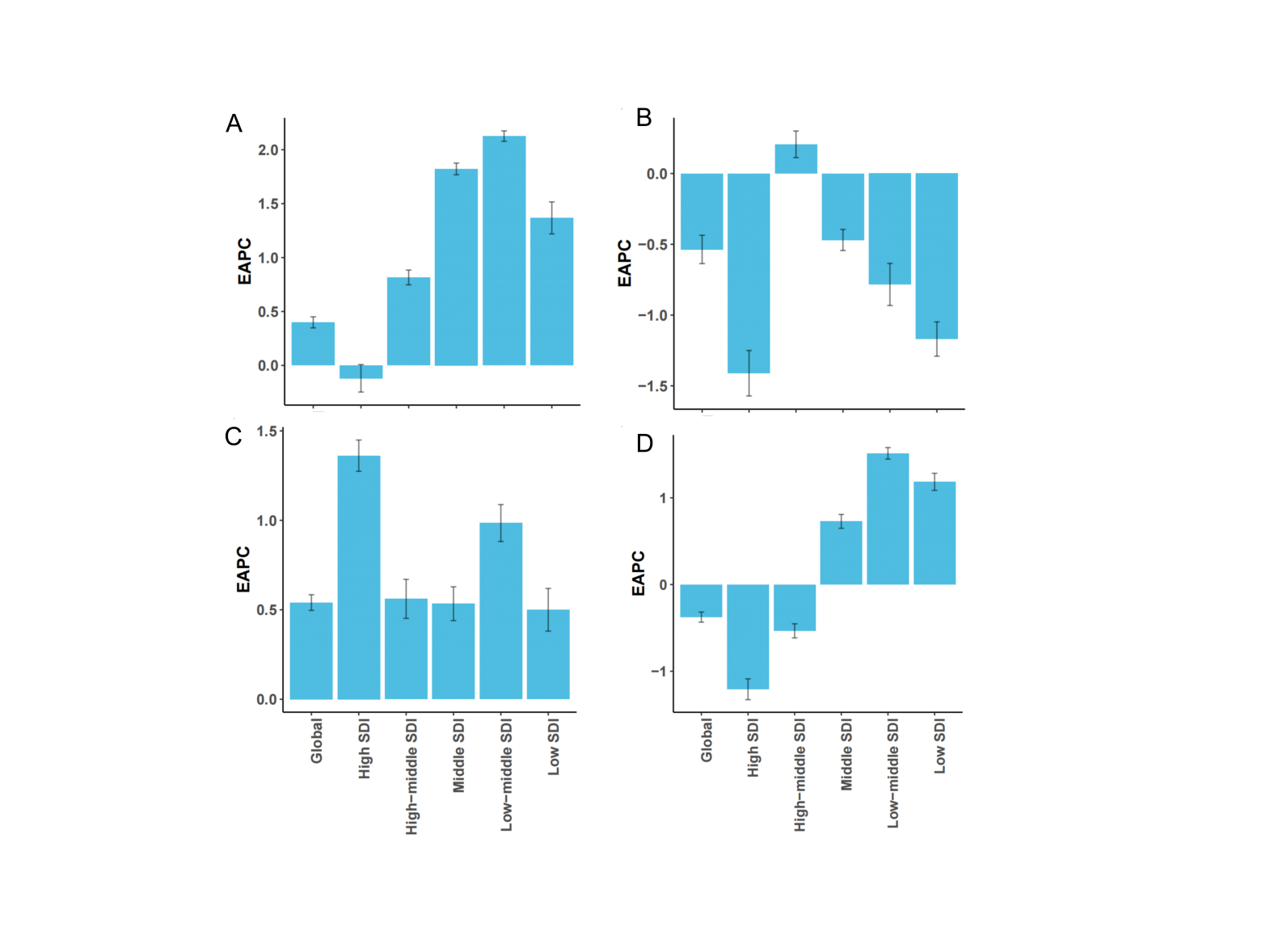


Supplementary Figure 1. ASIR of four types of female cancers by EAPC in different regions with low to high SDI. A is Breast cancer; B is Cervical cancer; C is Uterine cancer; D is Ovarian cancer. ASIR, age-standardized incidence rat; SDI, Socio-demographic index; EAPC, **estimated annual percentage change.**


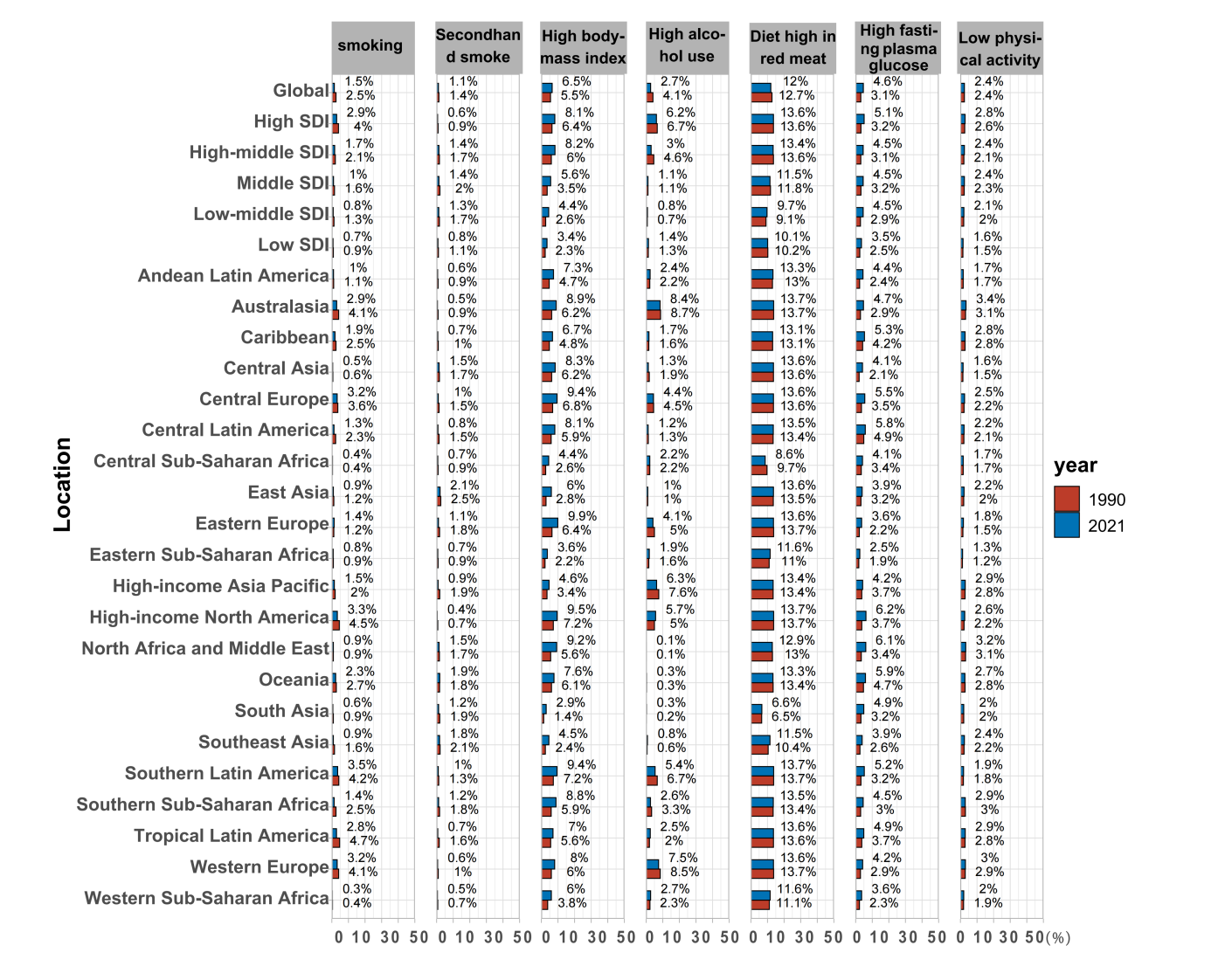
Supplementary Figure 2. Proportion of breast cancer deaths attributable to risk factors in regions with low to high SDI and across 21 GBD regions in 1990 and 2021. SDI, Socio-demographic index; GBD, Global Burden of Disease


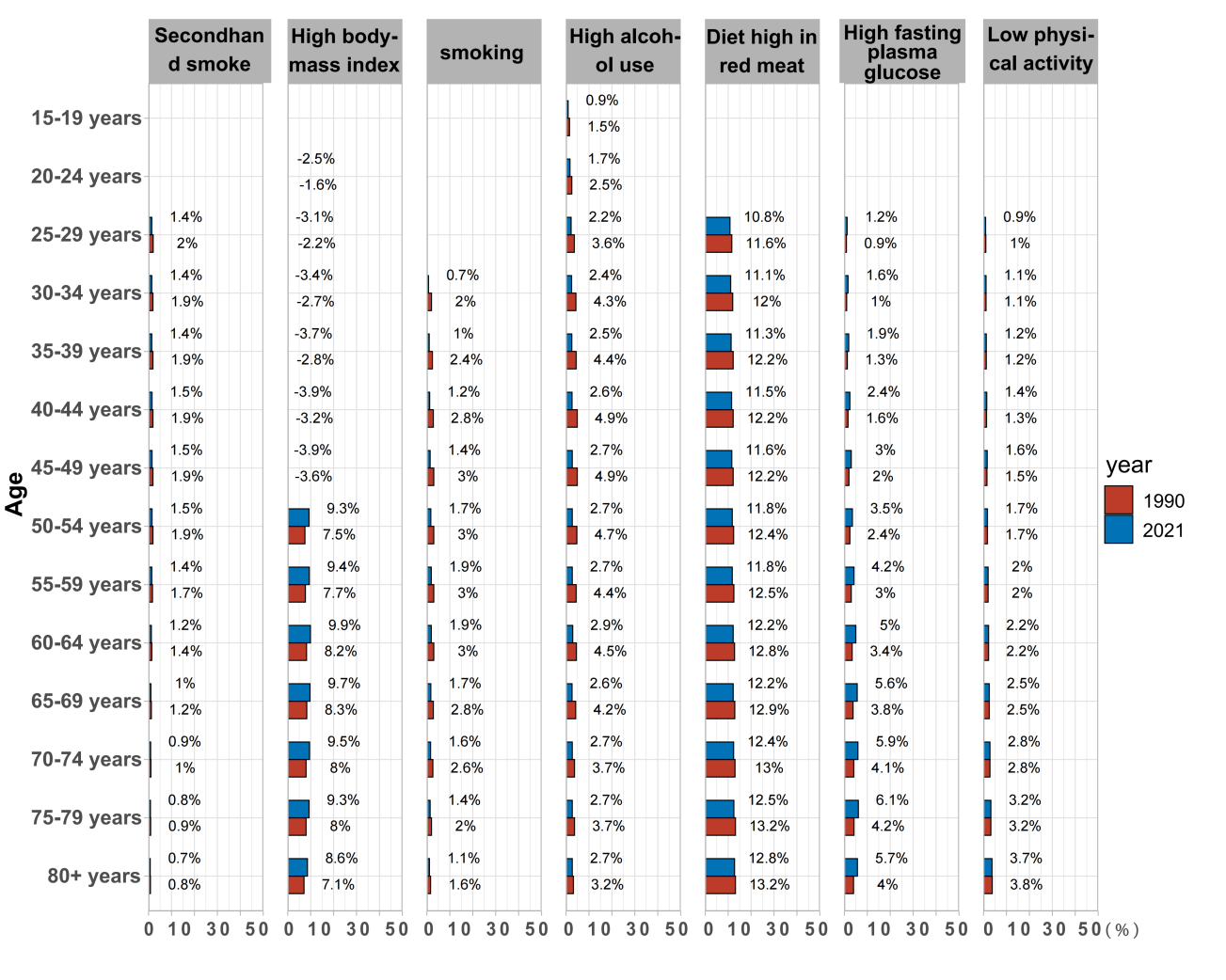


Supplementary Figure 3. Percentage of breast cancer-related deaths attributable to risk factors across different age groups in 1990 and 2021


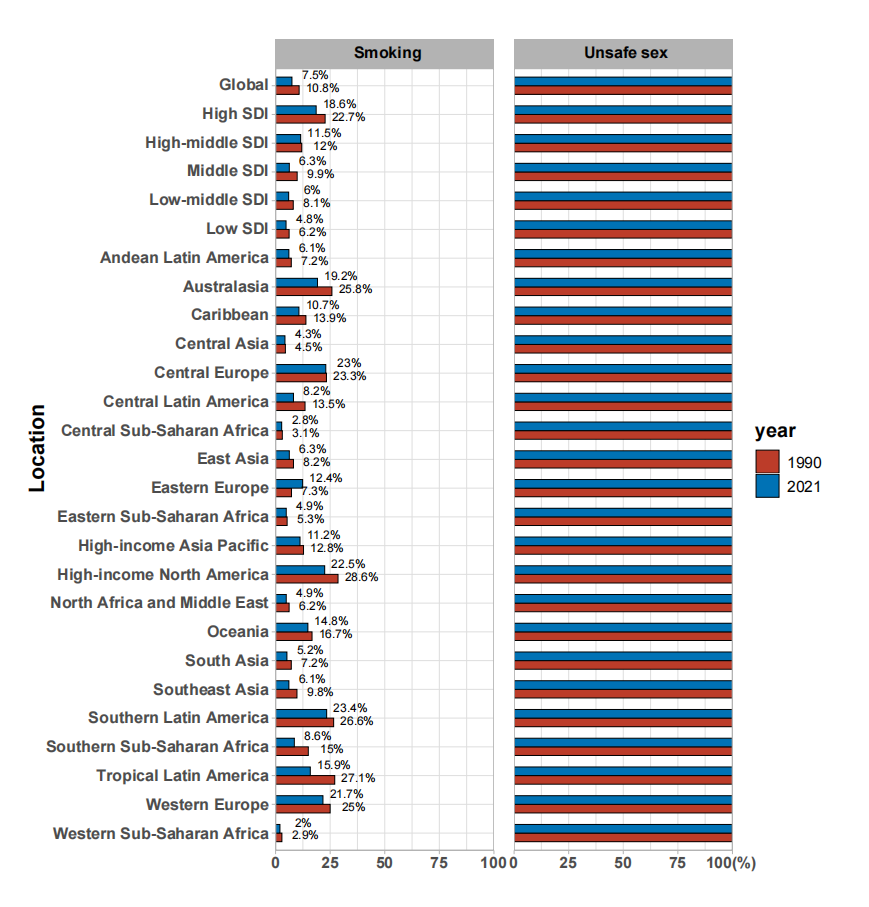


Supplementary Figure 4. Proportion of cervical cancer deaths attributable to risk factors in regions with low to high SDI and across 21 GBD regions in 1990 and 2021. SDI, Socio-demographic index; GBD, Global Burden of Disease


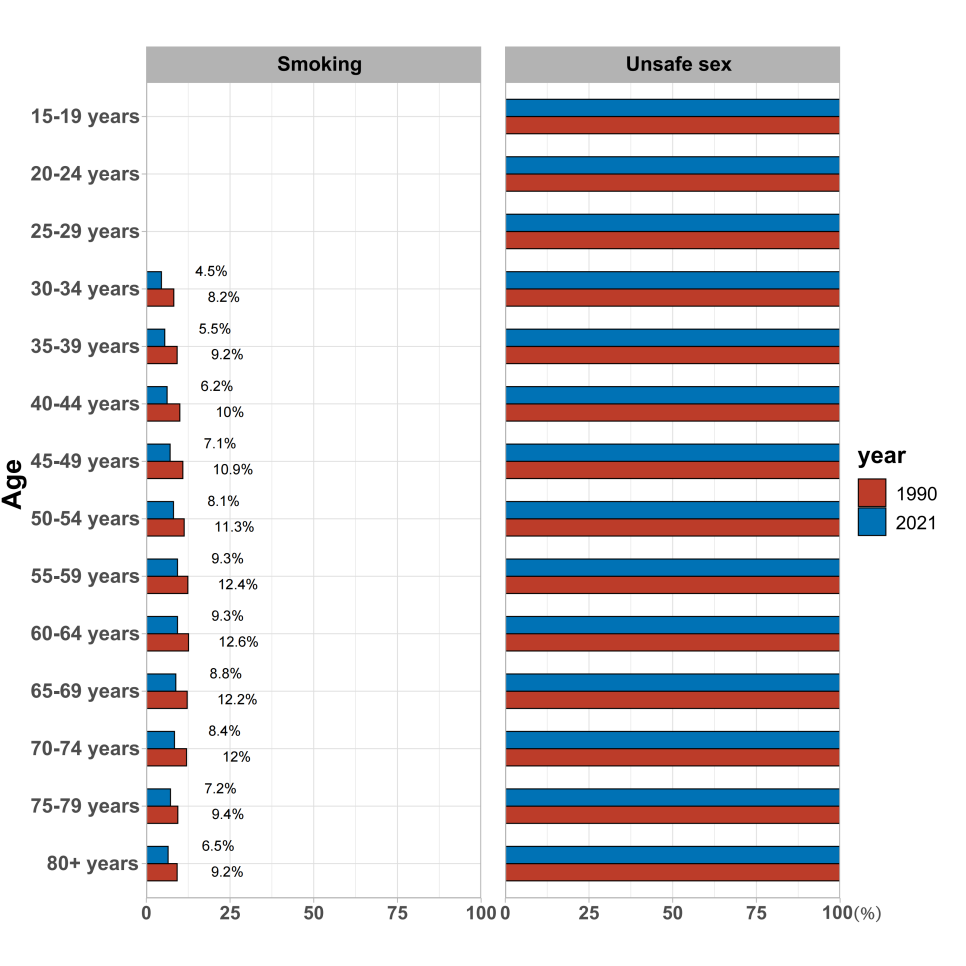


Supplementary Figure 5. Percentage of cervical cancer-related deaths attributable to risk factors across different age groups in 1990 and 2021


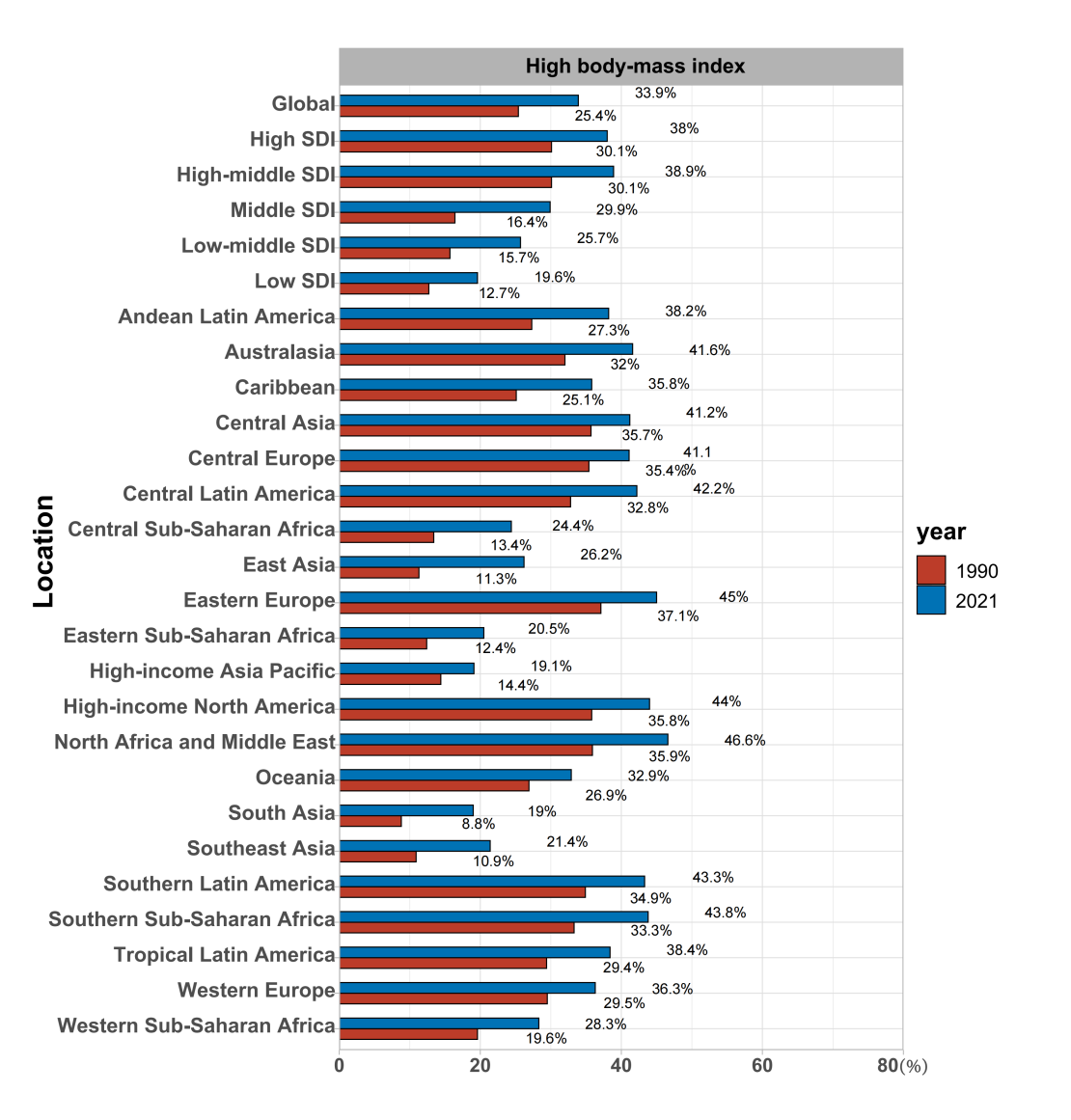


Supplementary Figure 6. Proportion of uterine cancer deaths attributable to risk factors in regions with low to high SDI and across 21 GBD regions in 1990 and 2021. SDI, Socio-demographic index; GBD, Global Burden of Disease


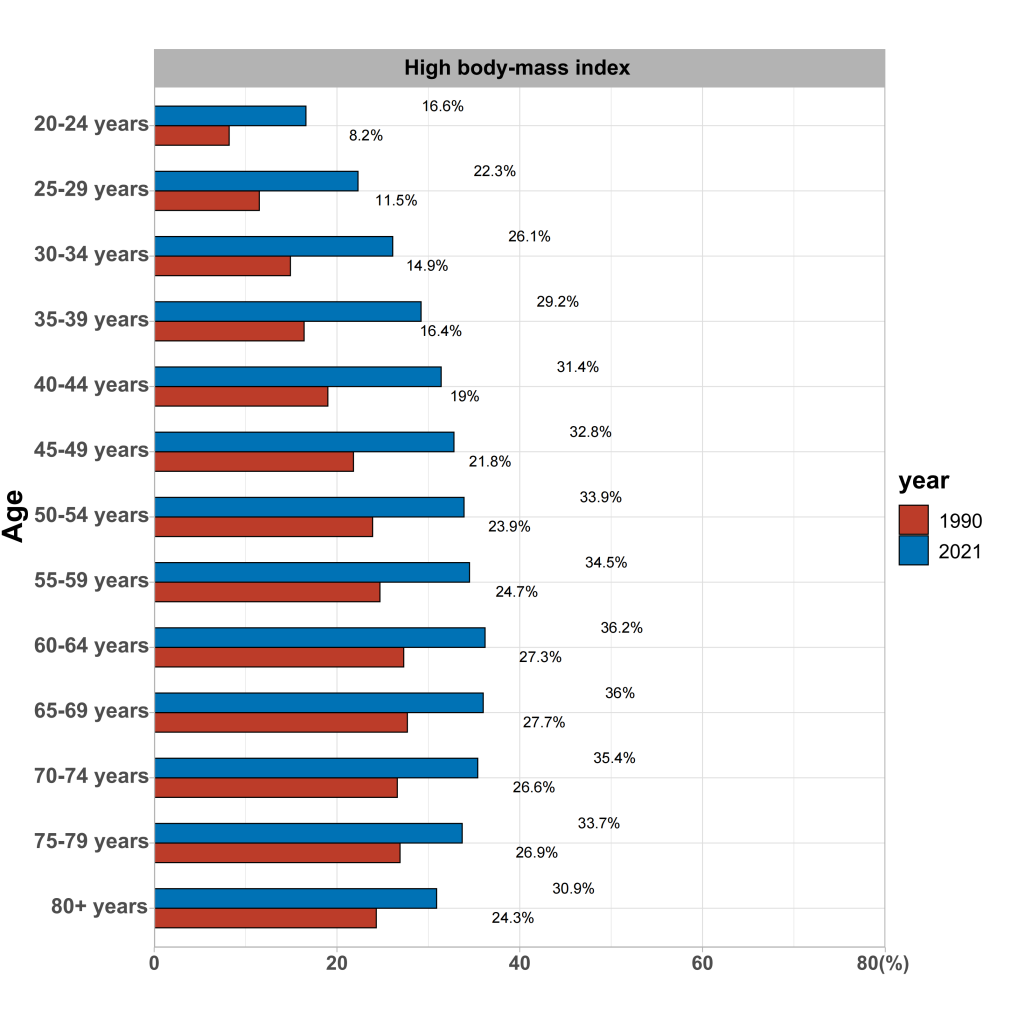


Supplementary Figure 7. Percentage of uterine cancer-related deaths attributable to risk factors across different age groups in 1990 and 2021


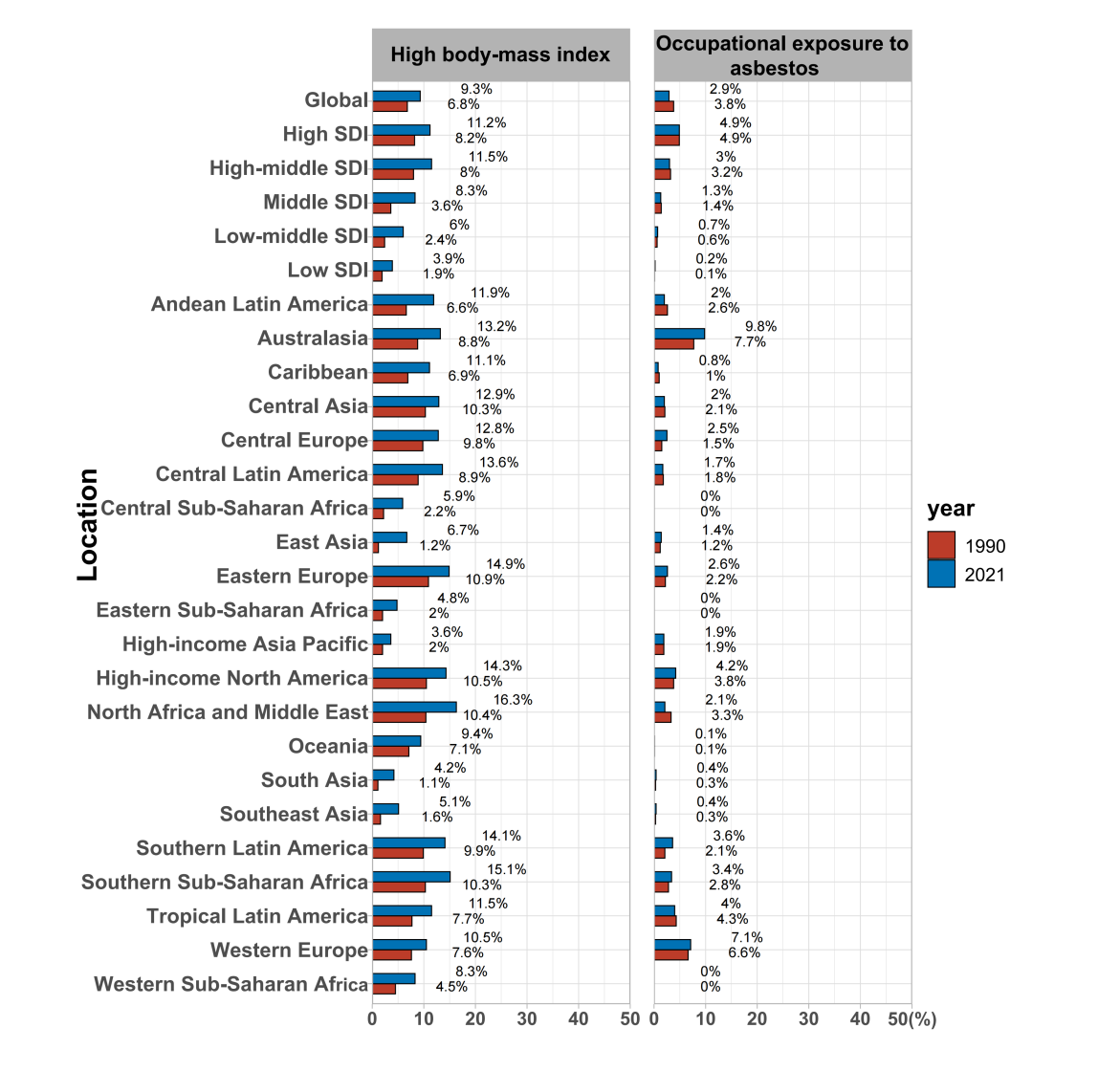


Supplementary Figure 8. Proportion of ovarian cancer deaths attributable to risk factors in regions with low to high SDI and across 21 GBD regions in 1990 and 2021. SDI, Socio-demographic index; GBD, Global Burden of Disease


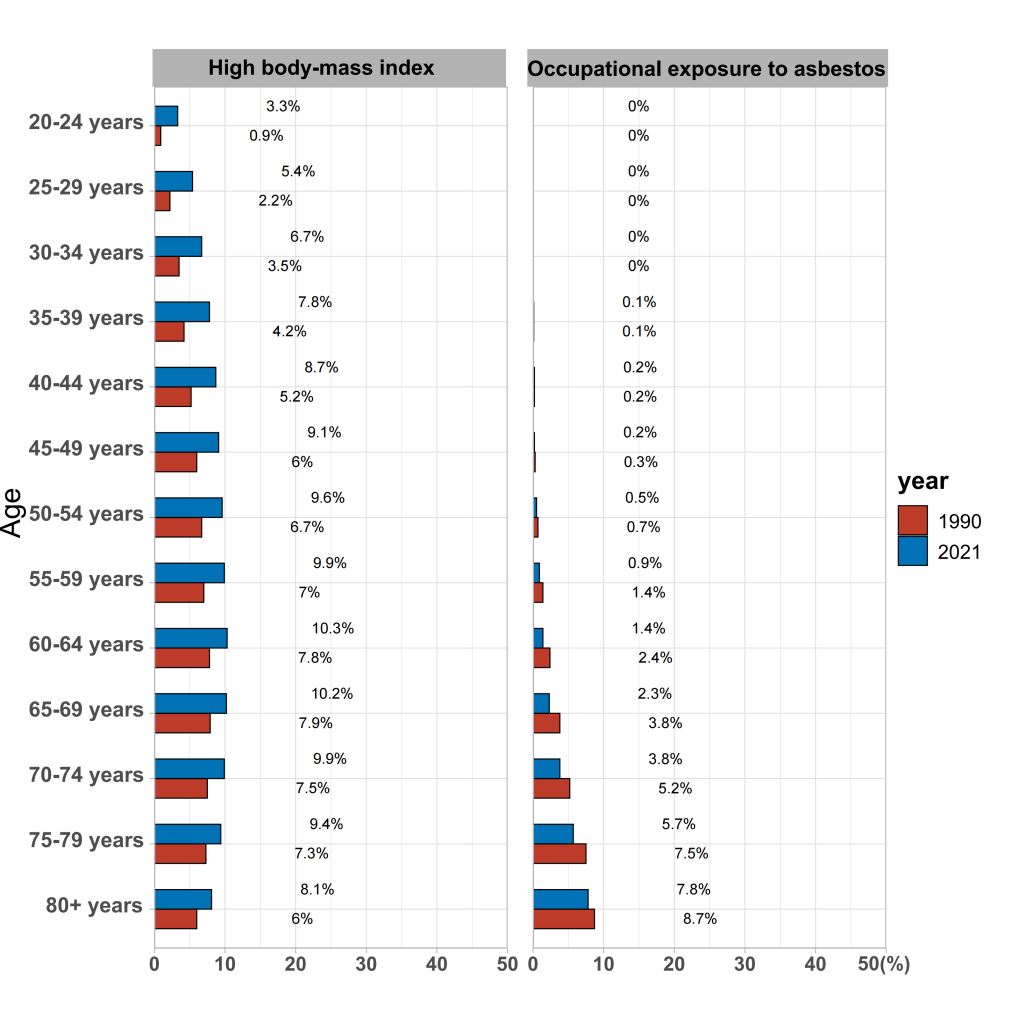


Supplementary Figure 9. Percentage of ovarian cancer-related deaths attributable to risk factors across different age groups in 1990 and 2021
